# High-Performance Piezoresistive MEMS Strain Sensor with Low Thermal Sensitivity

**DOI:** 10.3390/s110201819

**Published:** 2011-01-31

**Authors:** Ahmed A. S. Mohammed, Walied A. Moussa, Edmond Lou

**Affiliations:** 1 Mechanical Engineering Department, University of Alberta, Edmonton, AB, T6G 2G8, Canada; E-Mail: walied.moussa@ualberta.ca; 2 Department of Electrical and Computer Engineering, University of Alberta, Edmonton, AB, T6G 2V4, Canada; E-Mail: elou@ualberta.ca

**Keywords:** strain sensor, piezoresistive, MEMS, silicon

## Abstract

This paper presents the experimental evaluation of a new piezoresistive MEMS strain sensor. Geometric characteristics of the sensor silicon carrier have been employed to improve the sensor sensitivity. Surface features or trenches have been introduced in the vicinity of the sensing elements. These features create stress concentration regions (SCRs) and as a result, the strain/stress field was altered. The improved sensing sensitivity compensated for the signal loss. The feasibility of this methodology was proved in a previous work using Finite Element Analysis (FEA). This paper provides the experimental part of the previous study. The experiments covered a temperature range from −50 °C to +50 °C. The MEMS sensors are fabricated using five different doping concentrations. FEA is also utilized to investigate the effect of material properties and layer thickness of the bonding adhesive on the sensor response. The experimental findings are compared to the simulation results to guide selection of bonding adhesive and installation procedure. Finally, FEA was used to analyze the effect of rotational/alignment errors.

## 1. Introduction

New advances in the field of Micro Electro Mechanical Systems (MEMS) have broadened considerably the applications of these devices [1–3]. MEMS technology has also enabled the miniaturization of the devices, and a typical MEMS sensor is at least one order of magnitude smaller compared to a conventional sensor that is used to measure the same quantity. Consequently, MEMS devices can be patch-fabricated, which offers a high potential for cost reduction per unit. Moreover, proper design can solve some problems related to power consumption, while providing improved performance characteristics, such as accuracy, sensitivity and resolution.

Different sensing phenomena have been explored to develop MEMS sensors. These phenomena include modulation of optical [4–6], capacitive [7,8], piezoelectric [9], frequency shift [10] and piezoresistive properties [11–15]. Piezoresistive transduction has proved to have better performance compared to other sensing physics [16–18]. Moreover, the corresponding devices can overcome technical challenges related to chip integration; however, the response of piezoresistive devices under varying temperature conditions has limited their applications. Therefore, during the design and implementation of MEMS piezoresistive sensors, these shortcomings have to be considered.

It is well known that increasing dopant concentration reduces the sensor thermal drift [19–32] by stabilizing the values of the piezoresistive coefficients. On the other hand, the increase in dopant concentration also decreases the sensor sensitivity significantly. Another limitation during the application of the MEMS strain sensors is the signal loss resulted from the stiffness discontinuity when mechanical strain transmits through different structural layers, e.g., silicon carrier, bonding layer, *etc.* [11]. To account for this strain field alteration, multi-stage calibration and characterization processes have to be developed. In this sense, Finite Element Analysis (FEA) provides a reliable tool to carry out the required parametric studies in order to optimize the sensor performance.

In this work, a new piezoresistive MEMS strain sensor is introduced. The developed MEMS-based sensor has better performance characteristics compared to conventional thin-foil strain gauges, which demonstrates it as a potential candidate in structural health monitoring (SHM) applications. The chips incorporate piezoresistive sensing elements to measure mechanical strain via the observed changes in their resistivity or mobility. Five different doping concentrations were studied to cover low, medium and high doping levels. The fabricated chips were characterized over a temperature range from −50 °C to +50 °C. The effect of both geometrical and microfabrication parameters on the output signal strength was investigated.

The application range of the sensor is mainly restricted by both the electrical and mechanical properties of silicon crystal. single crystal silicon has better mechanical properties compared to other sensing materials [33–35]. FEA software was employed to investigate the potential rotational errors that can occur during the sensor installation and fabrication. The strain sensing chips were designed and prototyped bearing in mind flip chip packaging scheme, which permits subsequent integration with components of SHM systems. This work confirmed the feasibility of using high doping concentrations to realize high-performance piezoresistive MEMS sensors with acceptable sensitivity and stable thermal behavior.

## 2. Sensor Design and Modeling

Due relatively small magnitudes, π_11_ and π_12_ in p-type silicon are difficult to measure accurately. Published literature [22] indicates that values of these coefficients reported by different researchers have large discrepancies. The value of π_44_ is more consistent and relatively easy to measure. Moreover, at constant doping level, it is documented that π_44_ is independent of temperature [36,37]. Therefore, a piezoresistive sensor with output signal proportional to the shear piezoresistive coefficient (π_44_) will potentially have low thermal drift. The temperature effect contributes to the output signal of piezoresistive sensors through two sources: temperature coefficient of resistance (TCR) and dependence of piezoresistive coefficients on temperature. These two sources can be addressed by controlling the microfabrication parameters. The following sections discuss the formulation of piezoresistive sensor, sensing chip design and FEA modeling.

### 2.1. Formulation of Sensor Response

In the case of semiconductors, Ohm’s Law can be expressed as:

(1) $$E_{i}=\rho_{\mathrm{ij}}\;J_{j}$$

where *E_i_* and *J_i_* are the Cartesian vector components of electric field and current density, respectively, *ρ_ij_* is the electrical resistivity tensor [*ρ_ij_* = *ρ^o^*(*δ_ij_ + π_ijkl_*σ_*kl*_ + Λ_*ijklmn*_σ_*kl*_σ_*mn*_ + …)], *ρ^o^* is the electrical resistivity for the unstressed conductor filament, δ is Kronecker delta tensor and *π_ijkl_*, Λ_*ijklmn*_ …*etc.* are the components of fourth, sixth and higher order of piezoresistivity tensors, which describe the resistivity change due to the applied stress.

When the semiconductor piezoresistive element is subjected to stress or strain, the resistivity components are linearly related to the stress components by:

(2) $$\rho_{\mathrm{ij}}=\rho_{\mathrm{ij}}^{o}+\pi_{\mathrm{ijkl}}\;\sigma_{\mathrm{kl}}$$

**Figure 1.**
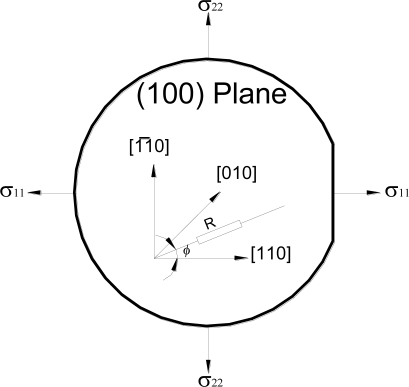
A piezoresistive element on (100) silicon substrate along general orientation (ϕ) with respect to [110] while subjected to biaxial state of stress.

Considering the case of biaxial state of stress, shown in Figure 1, a p-type piezoresistive element with orientation angle *ϕ* with respect to [110] direction will experience a normalized resistance change that can be described by:

(3) $$\begin{array}{c} \frac{\Delta R}{R}=\sigma_{11}\left(\frac{\pi_{11}+\pi_{12}+\pi_{44}\;\text{cos}\;(2\phi )}{2}\right)+\sigma_{22}\left(\frac{\pi_{11}+\pi_{12}-\pi_{44}\;\text{cos}\;(2\phi )}{2}\right)\\ +\sigma_{33}\pi_{12}+\sigma_{12}\;\left(\pi_{11}-\pi_{12}\right)\;\text{sin}\;(2\phi )+\left(\alpha_{1}T+\alpha_{2}T^{2}+....\right) \end{array}$$

where *T* is the difference between the operating temperature (*T_w_*) and the reference temperature (*T_o_*), and *π_ij_* are the temperature-dependent on-axis piezoresistive coefficients. *π_ij_* are related to *T* according to:

(4) $$\pi_{\mathrm{ij}}=\pi_{\mathrm{ij}}^{\left(o\right)}+\beta_{\mathrm{ij}}^{\left(1\right)}T+\beta_{\mathrm{ij}}^{\left(2\right)}T^{2}+....$$

where the terms (*α_1_T + α_2_T^2^* *+α_3_T^3^* + *….*) in Equation (3) account for the TCRs of the piezoresistive element. It is reported [20,22,32] that the first order TCR (*α_1_*) has a higher influence on the thermal response of piezoresistors than higher order TCRs (*α_2_, α_3_* *,….*). Moreover, it was determined that *α_1_* is the same for different crystal orientations [36,37]. In addition, in the case of heavily doped piezoresistors, ( $\beta_{ij}^{\left(1\right)}$, $\beta_{ij}^{\left(2\right)}$, …) have a minor contribution to the results [20,22,30–32].

### 2.2. Sensing Chip Design

**Figure 2.**
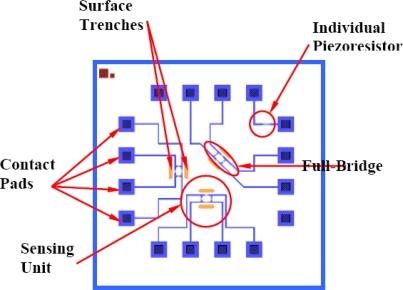
Schematic of the sensing chip design as shown on the microfabrication mask.

The current sensor design, shown in Figure 2, utilizes a sensing arrangement that is called sensing unit. The sensing unit is composed from four piezoresistive elements. The sensing chips have three sensing units; 0°, 45° and 90°. The 0° and 90° ones are utilized to measure two stress components while the 45° unit was implemented to measure the shear stress component; however, the output signal was very weak. The 0° and 90° units have sensing elements that are oriented along [110] direction and its in-plane transverse in a full-bridge configuration. The full-bridge arrangement reduces the sensor thermal drift by balancing of the effect of *α_1_* for different orientations. This process is highly dependent on the original values of the individual resistors.

**Figure 3.**
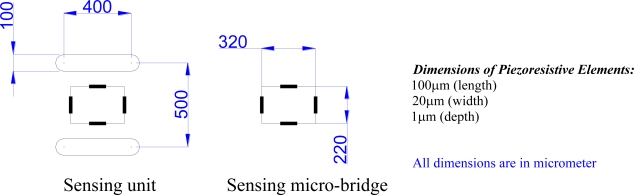
Dimensions of the sensing unit, sensing micro-bridge, and sensing piezoresistor.

**Figure 4.**
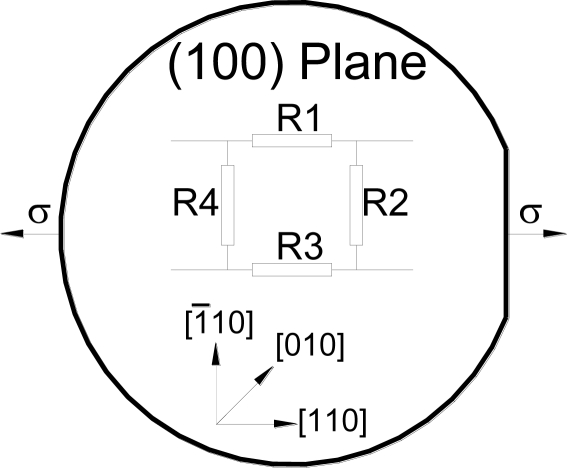
Schematic of the piezoresistive elements arrangement on (100) silicon substrate.

To improve the sensor signal strength, two grooves are etched parallel to the sensing direction, which defines the sensing unit. The dimensions of the sensing unit are shown in Figure 3. In addition to acting as stress risers to alter the stress field within the sensing unit, the surface grooves reduce the sensor cross-sensitivity, *i.e.*, the state of stress within the sensing unit is nearly uniaxial. Hence, the sensing unit can be considered subjected to uniaxial stress (σ), as shown in Figure 4. Therefore, the normalized resistance change of a full-bridge configuration can be calculated using Equation (3) for *ϕ* = 0° and *ϕ* = 90° yielding:

(5) $${\frac{\Delta R}{R}|}_{\begin{array}{l} \mathrm{full}\\ \mathrm{bridge} \end{array}}=2\pi_{44}\;\sigma$$

For input voltage (*V_i_*), the output voltage (*V_o_*) is expressed as a function of the normalized resistance change by multiplying Equation (5) by *V_i_* as:

(6) $${V_{o}|}_{\begin{array}{l} \mathrm{full}\\ \mathrm{bridge} \end{array}}=2\pi_{44}\;\sigma V_{i}$$

Examining Equations (5) and (6) shows that α_1_*T* is neglected due to the full-bridge effect. Equation (6) does not show the temperature dependency of the sensor output. However, the temperature effect is included in *π_44_* [according to Equation (4)], which is dependent on operating temperature and doping level of the piezoresistors [23]. Moreover, this equation applies to a flat sensing chip. Therefore, the effect of the surface grooves has to be evaluated experimentally or using FEA method. To neglect the effect of α_1_*T*, the microfabrication process has to be controlled to yield single piezoresistors with equal α_1_.

As shown in Figure 2, the sensing chip is a 10 mm × 10 mm square. The sensor silicon carrier is n-type silicon and the sensing elements are p-type silicon. The prototyping process of the sensor utilizes five different doping concentrations; 1 × 10^18^ atoms/cm^3^, 5 × 10^18^ atoms/cm^3^, 1 × 10^19^ atoms/cm^3^, 5 × 10^19^ atoms/cm^3^ and 1 × 10^20^ atoms/cm^3^. This range is used to evaluate the sensor performance at different doping levels and to account for variations during the microfabrication process. In addition, individual piezoresistive elements are included to facilitate the sensor characterization.

### 2.3. Finite Element Modeling

Finite Element Analysis (FEA) method is used to analyze the key parameters that can affect the sensor performance. The analyzed parameters are related to geometry (trenches’ dimensions, sensing element orientation…*etc.*), operation and installation (adhesive material properties and layer thickness, rotational error…*etc.*), and microfabrication (doping level, mask alignment error…*etc.*). Further details of the FEA modeling process can be found in the previous study [14]. The FEA models are divided into three groups. The first group is a flat (un-featured) 10 mm × 10 mm square sensing chip with four piezoresistive elements. The sensing elements are connected in a full-bridge configuration similar to the layout shown in Figure 4. This group is used for two purposes. The first purpose is to examine the reliability and accuracy of the FEA modeling process and the second purpose is to set reference values for subsequent analysis. The second group of FEA models has surface features (trenches or grooves), which is employed to quantify the stress concentration effect. The last group, full FEA model, includes the featured sensing chip in addition to bonding adhesive layer and strained surface. Details of the FEA model are presented in Table 1.

**Figure 5.**
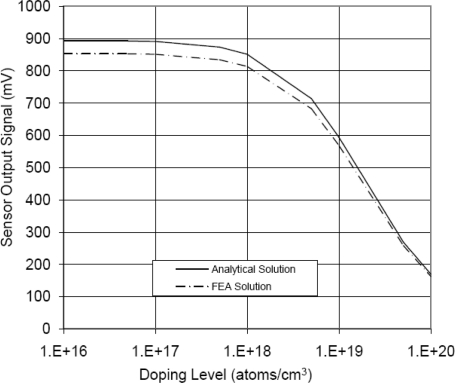
Comparison between FEA results and analytical results of flat sensing chip with full-bridge configuration at room temperature (25 °C) for light doping concentrations (less than 5 × 10^18^ atoms/cm^3^), V_i_ = 5 V.

**Figure 6.**
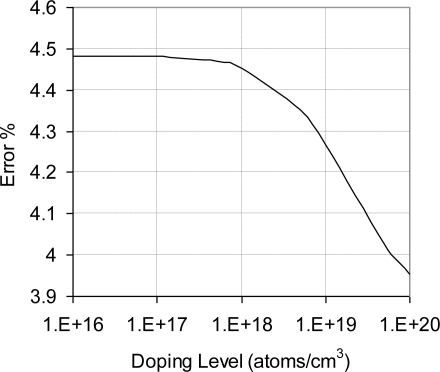
Error between the results of FEA model and analytical model of flat sensing chip with full-bridge configuration, V_i_ = 5 V.

The results from the flat sensing chip (first model) are compared to the analytical results from Equation (6) in Figure 5. It can be seen that as doping concentration increases, the difference between the FEA and the analytical solutions decreased. Both analytical and FEA solutions have the same general trend as a function of doping concentration; however, it was found that analytical solution has slightly higher values. This can be due to the impeded assumptions in the governing equations of the utilized element types in the FEA model, *i.e.*, some equations underestimate or overestimate the piezoresistive response depending on the doping level. Another reason for the slight discrepancy between FEA and analytical solutions is the mesh size of the FEA model. The % error was calculated in Figure 6. The % error is defined as the % difference between FEA solution and analytical solution divided by the analytical solution.

The trend in Figure 6 confirms that the reason of the difference between analytical and FEA solutions is the impeded assumptions in the governing equations of the element type rather than mesh size of the FEA model.

**Figure 7.**
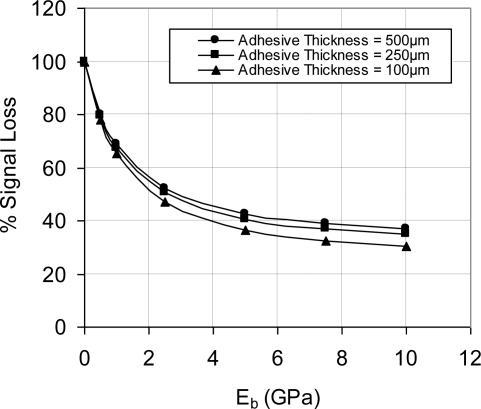
Effect of adhesive material properties (modulus of elasticity, E_b_) on the sensor output signal, V_i_ = 5 V.

**Figure 8.**
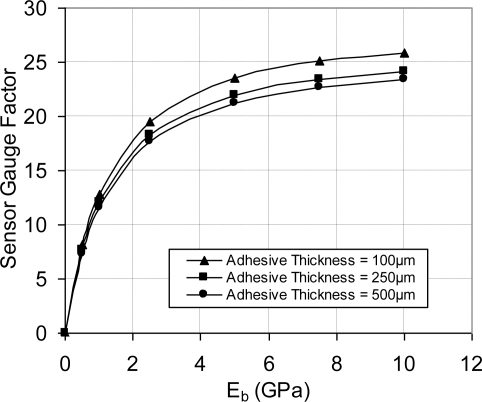
Effect of adhesive material properties (modulus of elasticity, E_b_) on the sensor gauge factor, V_i_ = 5 V.

This was examined by refining the mesh further; however, the same trend was found, *i.e.*, analytical solution is higher than FEA solution. The maximum % error (at light doping concentration) was less than 5% error decreased as the doping concentration increased. Bearing in mind that high doping level is favorable under varying temperature conditions, the FEA modeling procedure was considered highly descriptive to the analytical model and later to the fabricated sensing chips. As a result, the flat FEA model was geometrically modified to capture the geometric characteristics (features) of the sensing chip. Another key parameter that was considered in FEA simulation is the effect of bonding material on the sensor signal loss. The signal loss is mainly dependent on the modulus of elasticity and the layer thickness of the used adhesive. Therefore, a parametric FEA study was performed to guide in the selection of the boding adhesive and to help developing appropriate installation procedure. FEA was employed to evaluate the signal loss and the change in gauge factor using different modulus of elasticity and adhesive layer thickness. The results of this investigation are shown in Figures 7 and 8. It is noticed that at low modulus of elasticity, the effect of layer thickness is minor. However, as the modulus of elasticity increases, *i.e.*, the adhesive becomes stiffer; the adhesive layer thickness has major influence.

## 3. Sensor Prototyping

**Figure 9.**
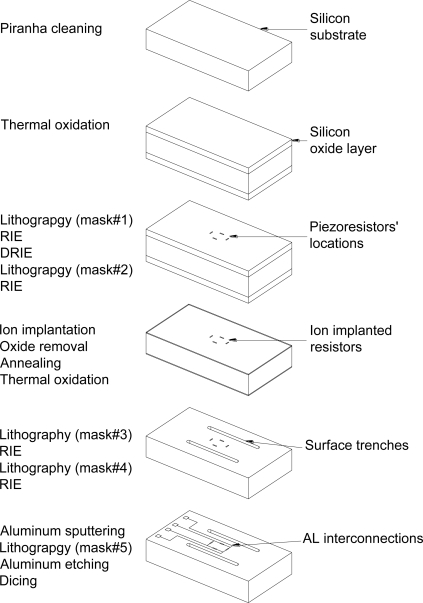
Microfabrication process flow to build the sensing unit.

To prototype the sensor, a five-mask microfabrication process flow based on bulk silicon micromachining was constructed. The microfabrication process utilizes 4-inch (100) n-type double side polished silicon substrates with primary flat along [110] direction. The wafer had thickness of 500 ± 25 μm, bulk resistivity of 10 Ωcm and total thickness variation less than 1 μm. The microfabrication process flow is shown in Figure 9.

It starts by wafer cleaning in piranha solution (3 parts of H_2_SO_4_ + 1 part of H_2_O_2_). Then, the following fabrication steps are applied:

1. Wet thermal oxidation to grow 1,200 nm of thermal oxide at 1,000 °C for 8 h in wet N_2_ atmosphere.
2. Lithography to pattern the first mask (alignment marks).
3. Reactive Ion Etching (RIE) then Deep Reactive Ion Etching (DRIE) to pattern the first mask in the silicon substrate.
4. Lithography to define the piezoresistors’ locations using the second mask (doping windows).
5. Boron ion-implantation with different doses (5.20 × 10^12^, 5.20 × 10^13^, 5.20 10^14^, 5.20 10^15^ and 5.20 × 10^16^ atoms/cm^2^) at energy level of 100 keV to create the p-type piezoresistive elements.
6. Masking oxide layer removal using RIE.
7. Annealing at 1,100 °C for 15 minutes.
8. Wet thermal oxidation to grow an insulating oxide layer for one hour at 1,000 °C.
9. Lithography to pattern the contact via for the aluminum contacts using the third mask (contact via openings).
10. RIE to open contact via.
11. Lithography to pattern the surface trenches using the fourth mask (surface trenches).
12. RIE to remove oxide from backside of the silicon substrate.
13. DRIE to reduce the silicon wafer thickness.
14. Aluminum sputtering for 30 minutes to get aluminum layer of thickness 500 nm.
15. Lithography to define metallization traces and interconnects using the fifth mask (metallization and interconnections).
16. Aluminum etching.
17. Annealing at 450 °C for 20 minutes in dry N_2_ atmosphere.
18. Wafer dicing, preparation for wire bonding and testing.

## 4. Experimental Setup and Testing Procedure

**Figure 10.**
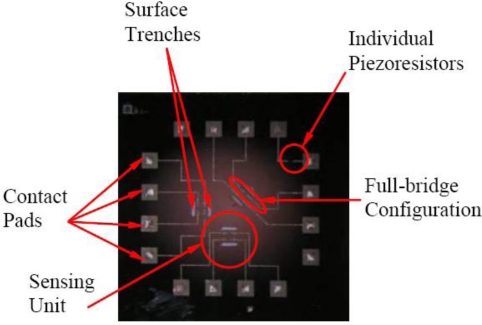
Fabricated sensing chip.

The fabricated sensing chip is shown in Figure 10. The experimental evaluation of the sensing chip involved construction of the I-V characteristic curve in addition to the evaluation of thermal properties (sensor TCRs), piezoresistive coefficient and stress/strain sensitivity. The following sections present the procedure to carry out the experimental evaluation of the fabricated sensing chips.

### 4.1. Testing Specimen Preparation

Test specimens were cut from cold rolled AISI 1020 steel long strips. The steel specimens had the following dimensions; length 405 mm, width 25 mm, and thickness 3 mm. The surface of the steel specimens was prepared for sensors installation. A 350 Ω thin-foil strain gauge from Vishay Instruments^®^ was installed on one side of the steel specimen in a quarter-bridge configuration and the required wiring was done. Moreover, a MEMS strain sensor was installed on the other side of the testing specimen. The used bonding adhesive in the installation process was M-Bond 200, which is a typical adhesive for thin-foil strain gauges. This bonding material proved to generate low stresses after curing at room temperature. To quantify the stress-induced due to adhesive curing, resistance of the piezoresistive sensing elements was measured before and after the sensing chip installation using a digital multimeter with resolution of 1 mΩ. The change of resistance due to adhesive curing was less than 0.005%, which is lower than reported values [22]. During mechanical testing, it was extremely difficult to collect the sensor output signal directly from the sensor contact pads while the cross-heads of the testing machine were moving. Therefore, special wiring process was designed to facilitate the signal transfer from the MEMS sensor to the data acquisition (DAQ) system.

**Figure 11.**
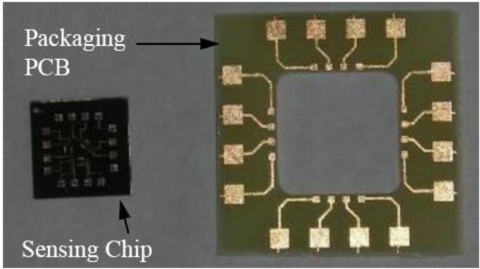
Testing printed circuit board and fabricated MEMS sensing chip before installation on the testing specimen.

**Figure 12.**
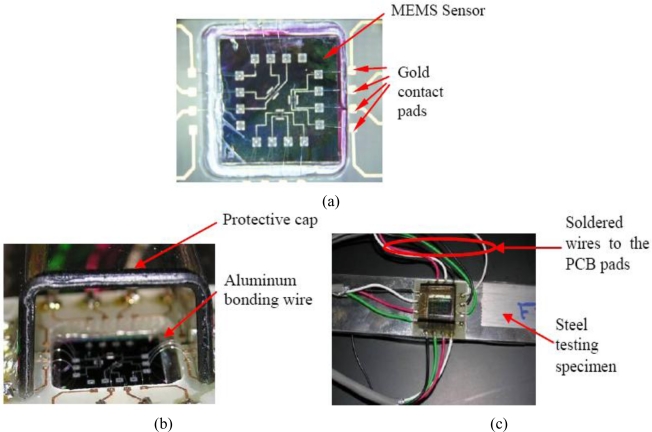
Prepared testing specimen; **(a)** after wire bonding, **(b)** after placing the polymeric protective cap and **(c)** top view of the wired specimens.

As a result, testing printed circuit board (PCB) was designed and constructed, shown in Figure 11. A PCB was bonded around the installed MEMS sensor. Wire bonding was performed to connect the MEMS sensor and the PCB terminals. Electrical wires were then soldered to the PCB pads. Finally, a polymeric cap was placed to prevent any potential damage to the testing PCB, MEMS sensor or the fine bonded wires. The prepared testing specimen is shown in Figure 12.

### 4.2. Calibration Procedure

The testing process started by initial resistance measurements in order to document the sensor readings at no load and zero input voltage. Then, the sensor I–V characteristic curve was constructed up to 8 volts. The main function of the I–V characteristic curve was to determine the suitable input voltage to operate the sensor within its linear range (if it exists). The sensor I–V characteristic curve was built on a forward-bias when junction voltage existed. Using an experimental environmental chamber, the sensor was subjected to different temperatures, from −50 °C to +50 °C with 25 °C interval, at no load. The resistance change in response to the surrounding temperature was recorded. Then, the normalized resistance change was calculated and plotted versus temperature. This step was performed to evaluate the sensor TCR, which represents the sensor sensitivity to temperature.

**Figure 13.**
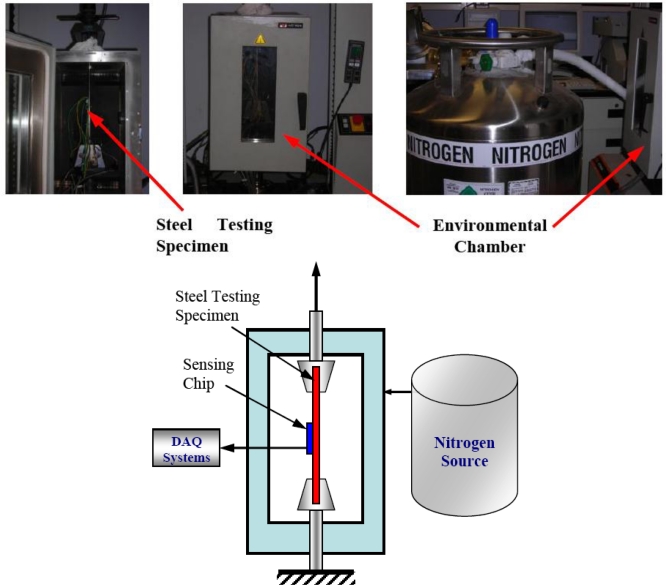
Testing system.

After TCR evaluation, the MEMS sensors were mechanically tested, according to ASTM E8 Standard [38], at different temperatures using the experimental setup shown in Figure 13. The load was applied to put approximately 1,500 με on the steel testing specimen. The maximum strain value was selected based on the testing machine capacity. The load was ramped from 0 to 25 kN over about 10 minutes. Load and stroke data were collected from the testing machine controller. Simultaneously, output signals from the thin-foil strain gauge and MEMS strain sensor were measured. The applied strain was confirmed using the thin-foil readings and the load data from the testing machine.

**Figure 14.**
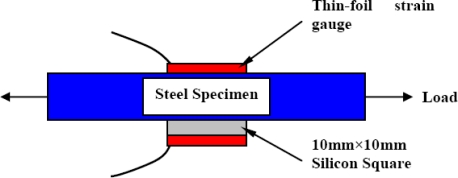
Schematic of the specimen to evaluate the relation between near-field strain and the far-field strain.

To quantify the signal loss due to the bonding adhesive and the silicon chip thickness, another testing specimen was prepared. On one side of this specimen, a 350 Ω thin-foil strain gauge was installed to measure the applied strain from the testing machine (far-field strain). On the other side of this specimen, instead of a MEMS sensor, a 10 mm × 10 mm silicon square was bonded to the steel specimen then another 350 Ω thin-foil strain gauge was bonded on top of the silicon square to measure the transferred strain through the bonding adhesive and the silicon chip after it undergone all of the signal losses, which was called near-field strain. A schematic of the used specimen to evaluate the strain field alteration is shown in Figure 14. Since the thin-foil strain gauge calibration curve is well known from the manufacturer datasheet, the installed thin-foil strain gauges provided the relationship between the far-field strain and near-field strain.

### 4.3. Data Analysis and Signal Processing

After the data was collected, the mean and the standard deviation of the measurements were calculated. Then, the following steps were carried out to process the output signal:

1. The sensor output signal was plotted as a normalized resistance change (ΔR/R) versus temperature at load-free condition.
2. The slope of (ΔR/R) versus temperature was evaluated based on linear regression model. This slope represents the average sensor TCR, which was calculated in parts per million per degree Celsius (ppm/°C).
3. Mechanical strain was calculated using the applied load and the steel testing specimen characteristics (dimensions and material properties).
4. The applied strain was verified using readings from the thin-foil strain gauge.
5. Temperature effect was removed from the sensor output signal using the evaluated TCRs.
6. The sensor output signal was plotted as output voltage versus strain to construct the sensor calibration curves.
7. Initial offset was removed from the sensor calibration curves.
8. Sensor sensitivity was evaluated by calculating slopes of the different calibration curves using linear regression model at different temperatures; −50 °C, −25 °C, 0 °C, +25 °C and +50 °C.

## 5. Results and Discussion

The ASTM E251-92 Standard [39] was adapted to evaluate the performance characteristics of the MEMS strain sensor. This Standard was originally developed to evaluate metallic bonded resistance strain gauges. Therefore, it was the most applicable Standard to compare the thin-foil gauge and the fabricated MEMS sensor. The following sections are dedicated to discuss different parts; I–V characteristic curves, far- and near-field strain relationship, sensor TCRs, calibration of the MEMS sensor at different temperatures, effect of geometric features, piezoresistive coefficients and rotational error analysis.

### 5.1. I-V Characteristics

**Figure 15.**
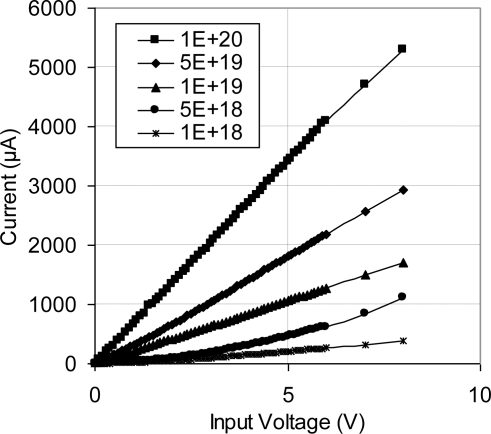
I-V characteristic curve of the developed MEMS sensor at room temperature. The linear relationship indicates good ohmic contact between aluminum metallization and p-type silicon.

The I-V characteristics curves of the fabricated sensing chips were constructed at room temperature. Figure 15 presents these curves for five doping concentrations up to 8 volts. The sensor I–V characteristics of lower concentrations started with curved portion followed by a straight line. This curved portion is a sign on diode junction existence. The initial curvature is more obvious as doping level decreased. The curves were constructed based on forward bias, when diode junction existed. High doping concentrations (5 × 10^19^ atoms/cm^3^ and 1 × 10^20^) have straight I–V curve, which confirms good ohmic contact between aluminum metallization and p-type silicon. The slope of these curves increased as the doping level increased, an indication on lower electrical conductivity, *i.e.*, increased resistivity. The constructed characteristics curves were utilized to determine the proper bridge input in order to operate the MEMS sensor in its linear range, which was selected as 5 V for all of the subsequent testing stages.

### 5.2. Far-Field and Near-Field Effects

Due to the mismatch of the mechanical properties between silicon and bonding adhesive (M-Bond 200), stress discontinuity was induced between the different structural layers. This stress discontinuity resulted in signal loss, which had to be quantified. The signal loss can be estimated either experimentally or using FEA. Figures 7 and illustrate the FEA version of this evaluation based on different adhesive layer thickness and various modulus of elasticity. However, to achieve this step experimentally, the specimen shown in Figure 14 was utilized.

**Figure 16.**
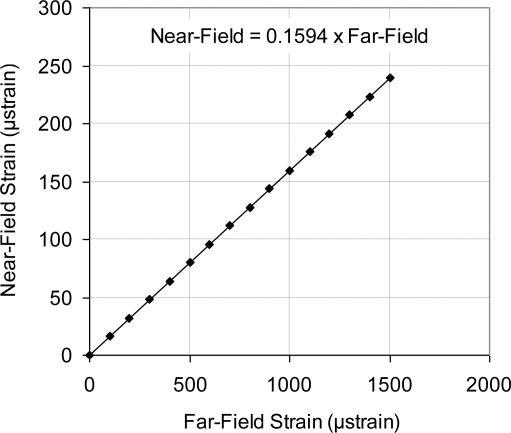
Relation between far-field strain and near-field strain. The transferred (near-field) strain is ∼16% from the applied (far-field) strain.

The relationship between the far-field and the near-field strains is plotted in Figure 16. The slope of this graph shows that approximately 16% of the applied mechanical strain on the steel testing specimen (far-field strain) was transferred through the bonding adhesive and the silicon carrier, which was then sensed by the piezoresistive elements (near-field strain). Similar behavior was reported [11] at higher signal transfer. In this work [11], the sensor was an integral part of composite material within its matrix, which lowered the signal loss. In the current sensor design, the signal is only transferred through one surface, lower surface of the sensing chip.

### 5.3. TCR Evaluation

The temperature coefficients of resistance (TCRs) of the fabricated piezoresistors were evaluated by subjecting the fabricated chips to different temperatures from −50 °C to +50 °C at load-free condition. The resistance values were measured. It was reported [22,40] that over temperature range from −150 °C to +125 °C (at doping level of about 2 × 10^18^ atoms/cm^−3^) the measured resistance was high at low temperature. Following to this high resistance, there was a monotonic decrease until the resistance reached a minimum value at approximately −45 °C. Then, the resistance starts to increase as the temperature increases. A comparison of the current experimental results to this work [22,40] showed good agreement over the same temperature range from −50 °C to +50 °C.

**Figure 17.**
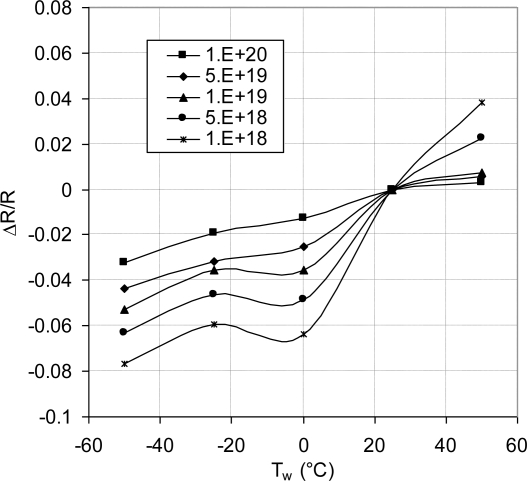
Temperature response of the sensing unit at stress-free condition for different doping concentrations to evaluate the sensor TCR.

In the case of low doping level, at low temperature, most of the charge carriers tend to freeze out onto donors and acceptors, which results in increased resistance at low temperatures. As the temperature increases the freeze out effect decreases until the resistance reaches a minimum value. This minimum value was reported to be at about −45°C [22,40] for doping concentration of about 2 × 10^18^ atoms/cm^−3^. Beyond this temperature, the absorbed thermal energy increases random scattering of the charge carriers and hence the electrical resistance increases at low doping concentrations. In case of medium to relatively high doping concentrations, the available number of charge carries can counterbalance the random scattering. Hence, the resistance value decreases or stays nearly constant. At extremely high doping concentrations, the random scattering is overcome and the piezoresistive element can act as linear resistor with ohmic behavior. This observation is confirmed by examining Figure 17 for doping concentration of 1 × 10^20^ atoms/cm^3^. During the sensing operation, applying electric field forces the charge carriers to move in the direction of current flow, which reduces the effect of the random scattering.

**Figure 18.**
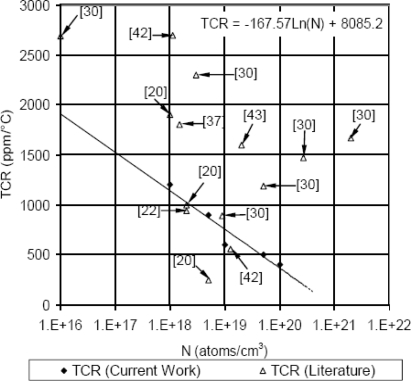
Temperature coefficient o**f** resistance (TCR) at different doping concentrations to evaluate the sensor TCR.

Referring to room temperature measurements, the normalized resistance change (ΔR/R) at load-free condition was plotted versus operating temperature as shown in Figure 17. The average slopes of the individual curves in this figure represent the combined average TCRs. This combined TCR is composed from first order, second order and higher order TCRs, which describes the thermal drift of the MEMS sensor. Moreover, it includes the local effect of the geometric features. The sensor TCRs were evaluated based on linear regression model. The sensor TCRs were then plotted as a function of doping concentration, as shown in Figure 18.

It was found that increasing doping concentration reduced the sensor TCR, which agrees with the published literature [22,27,30,31,40,41]. However, a work by Boukabache and Pons [20] showed that first order TCR decreased with doping level up to about 5 × 10^18^ atoms/cm^3^. Then, it increased as doping concentration increased (see Figure 1 in Reference [20]). The presented TCRs in Figure 18 are the combined effects of all orders TCRs in addition to the local effect of the geometric features. Values of TCRs were collected from literature [20,22,30,37,40,42] to perform further comparison. A similar procedure was used to calculate the TCRs from the published literature using the appropriate figures. A summary of the literature values TCRs is presented in Table 2.

For doping concentrations between 1 × 10^18^ atoms/cm^3^ and 1 × 10^19^ atoms/cm^3^, references [20,22,30] gave TCRs that are very close to the current work. Except for doping concentration of 5 × 10^18^ atoms/cm^3^ from reference [20], the current work has lower TCRs compared to the published literature. This can be explained by the combined effect of the full-bridge configuration that formed the sensing unit. The full-bridge acted to partially cancel out the effect of the TCRs of the individual piezoresistive elements. While the microfabrication process in the current work utilized ion implantation, some of the literature values were the results of diffusion and some were not clearly documented. Ion-implantation proved to provide more uniform properties compared to diffusion. Finally, introducing the geometric features in the sensor silicon carrier helped to reduce local thermal deformation. This last point is supported by Figures 22 and 24 in Reference [41], which show the effect of the trench layout on the overall senor TCR.

### 5.4. Sensor Calibration

The steel testing specimens were loaded using a universal testing machine equipped with an environmental chamber. The applied strain was calculated and compared to the measured values using thin-foil strain gauge. To deal with the fluctuations in readings, a statistical approach was adapted to calculate the average reading of the measurements. The sensor calibration curves were constructed using the applied strain (far-field strain) versus sensor output signal. Using the far-field strain to construct the calibration curves included the effect of bonding adhesive in measurements. Therefore, the calculated gauge factor and sensitivity were called equivalent gauge factor and equivalent sensitivity, respectively. The relationships between the equivalent parameters (gauge factor and sensitivity) and their corresponding piezoresistive values can be defined experimentally or through FEA. Figure 7 and Figure 8 present the FEA results and Figure 18 establishes this relationship experimentally.

**Figure 19.**
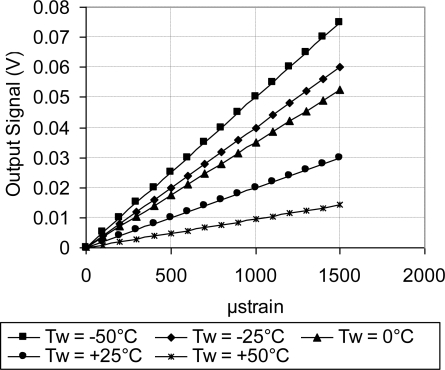
Sensor calibration curves at different operating temperatures for doping concentration of 1 × 10^18^ atoms/cm^3^, V_i_ = 5 V.

**Figure 22.**
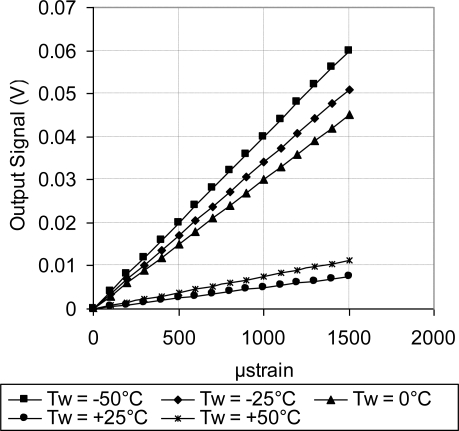
Sensor calibration curves at different operating temperatures for doping concentration of 5 × 10^19^ atoms/cm^3^, V_i_ = 5 V.

**Figure 24.**
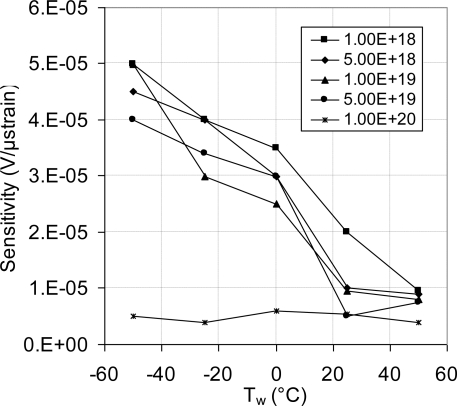
Temperature effect on the sensor sensitivity at different doping concentrations.

As discussed above, Figure 18 showed systematic decrease in the sensor TCR as the doping concentration increases. For example, at doping concentration of 1 × 10^20^ atoms/cm^3^, the TCR has dropped to about one third of its value at doping concentration of 1 × 10^18^ atoms/cm^3^. This drop in the sensor TCR helped to develop a MEMS piezoresistive sensor with low temperature drift; however, this improvement in the sensor TCR came on the expense of the sensor equivalent sensitivity as shown in Figures 19 through 23. As expected, the sensor output signal was found to follow linear relationship with the applied mechanical strain/stress. The sensor calibration curves shown in Figure 19 through 23, were constructed for various doping concentrations at different operating temperatures. Figure 24 summarizes the calibration results at different operating temperatures. Examining this figure demonstrated that high doping concentration helped to stabilize the sensor output signal, which can be depicted from the nearly horizontal line in Figure 24, which belongs to doping concentration of 1 × 10^20^ atoms/cm^3^. On the other hand, at low doping concentrations, due to the diode junction, the leakage current was temperature-dependent, which limits the capabilities of MEMS strain sensors under varying temperature conditions. Values of standard deviation of the calculated sensor sensitivity are listed in Table 3.

### 5.5. Effect of Geometric Features

**Figure 25.**
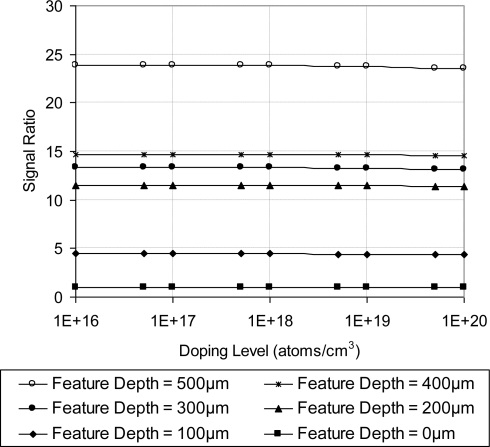
Effect of geometric feature depth on the sensor signal ratio (longitudinal sensitivity/transverse sensitivity) at different doping concentrations, V_i_ = 5 V. For 0 depth the signal ratio is unity.

The introduced geometric features (surface trenches) provided two valuable effects. First, through stress/strain concentration effect, they acted as stress/strain risers, which magnified the differential stress in the vicinity of the piezoresistive sensing elements. This magnification enhanced the output signal strength and hence sensitivity. Second, the surface trenches reduced the sensor cross sensitivity. These two functions were confirmed by FEA simulation of the featured sensing chip. Figure 25 shows that increasing the trench depth improves the signal ratio (longitudinal sensitivity to the transverse sensitivity). The flat sensing chip has signal ratio of unity. The simulated results are prepared at room temperature (25 °C). Moreover, it is clear that the signal ratio is independent of the doping concentration. Finally, the FEA simulation results were verified for feature depth of 100 μm, which showed that the output signal strength from the sensing unit that makes 0° with [110] is about one order of magnitude compared to the sensing unit that is 90° with the same crystallographic direction.

### 5.6. Piezoresistive Coefficients Evaluation

For any resistor orientation, by examining Equation (3), it is clear that applying a uniaxial stress along [110] direction or its in-plane transverse yields normalized resistance change that is a function of π_44_ and (π_11_ + π_12_). Therefore, only these coefficients can be measured individually using the loading case used in the current work. Applying hydrostatic pressure can provide enough information to evaluate all of the piezoresistive coefficients. However, applying hydrostatic pressure considerably complicates the calibration procedure.

**Figure 26.**
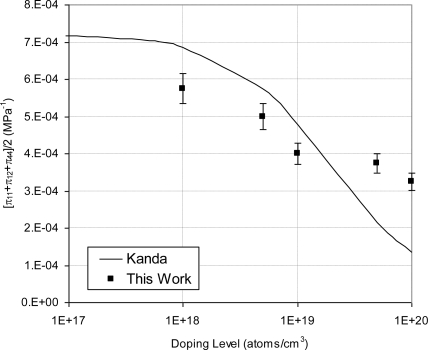
Comparison between experimental values of ([π_11_ + π_12_ + π_44_]/2) and Kanda’s model [23] at room temperature.

**Figure 27.**
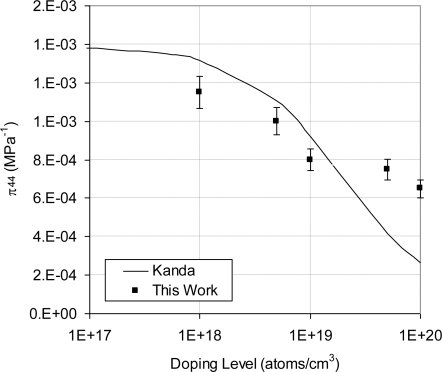
Comparison between experimental values of π_44_ and Kanda’s model [23] at room temperature.

Alternatively, a three-element off-axis rosette [37] can be used to evaluate the individual piezoresistive coefficients. The output signal from a flat sensing chip is proportional to ([π_11_+π_12_+π_44_]/2), which can be defined as the piezoresistive gauge factor. Moreover, the same flat sensing chip was used to extract π_44_ at room temperature. The experimental results of ([π_11_+π_12_+π_44_]/2) and π_44_ were compared to analytical model by Kanda [23] in Figure 26 and Figure 27, respectively. From these figures, it is clear that Kanda’s model gives good estimate of the piezoresistive gauge factor and the shear piezoresistive coefficient up to doping concentration of 1 × 10^19^ atoms/cm^3^. However, at higher doping concentrations it underestimates them. This observation agrees with the work by Harley and Kenny [44].

### 5.7. Effect of Alignment Errors

**Figure 28.**
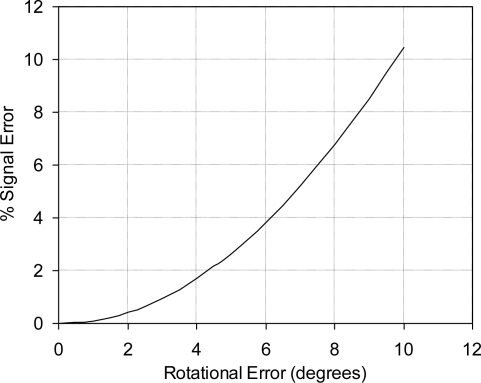
FEA results showing the effect of alignment/rotational error on the sensor output signal. The rotational error is measured from [110] direction, number of FEA runs = 16.

Successful application of piezoresistive sensors requires properly designed sensing chips and the awareness of potential sources of error during the sensor application. In particular, rotational alignment error, during fabrication and installation, can be considered one of the most important sources of errors. Another factor that can cause significant variability, when comparing results, is the purity of the used silicon substrates and the oxygen levels in the silicon samples. The effects of crystallographic misalignment and thermal errors were not mentioned in most of the published literature; however, Jaeger and Suhling [45] showed that temperature variations and measurement errors can play a pivotal role in determining accuracy of the results obtained during both calibration and application of piezoresistive stress sensors. Therefore, the goal of this section is to investigate the sensitivity of the fabricated sensing chip to alignment/rotational errors, which can affect the sensor output signal. FEA simulation was used to analyze the effect of rotational error on the sensor output signal. The alignment error around the center of the chip is plotted versus the % signal error in Figure 28. It is clear that about 4.5° alignment error can introduce error in the sensor output signal of about 2%, which is an acceptable value. Therefore, the current sensor design can be considered to have low sensitivity to rotational errors within ±4.5° misalignment. It is also noted that the induced error due to the rotational misalignment is non-linear.

## 6. Conclusions

In this work, a MEMS piezoresistive strain sensor that utilizes p-type sensing elements was successfully designed, fabricated and calibrated. A calibration technique for the MEMS strain sensor was described. Near-field and far-field strain concepts were discussed to account for signal loss. Moreover, the relationship between the far-field and the near-field strains was experimentally established. Approximately 16% of the applied mechanical strain on the strained surface (far-field strain) is transferred to the sensing elements (near-field strain). The ratio between the far-field to the near-field strain can be improved by one or more of the following actions; wafer thinning, introducing other features in the bottom surface of the sensing chip, and use of SOI wafer while etching the oxide layer underneath the sensing unit. To verify the FEA modeling process, FEA results were compared to the analytical solution of a flat sensing chip. The maximum % error (at light doping concentration) was less than 5% and the minimum % error (at high doping concentration) was approximately 2%. The experimental results showed that the sensor TCR at doping concentration of 1 × 10^20^ atoms/cm^3^ dropped to approximately 30% of its value at doping concentration of 1 × 10^18^ atoms/cm^3^. The overall sensor TCR followed a logarithmic relationship with the doping level.

It was proved that high-sensitivity MEMS piezoresistive strain sensor can be developed using high doping concentration, e.g., 1 × 10^20^ atoms/cm^3^. The average measured strain sensitivity using this doping concentration is 4.90 ± 0.894 μV/με at different temperatures, from −50 °C to +50 °C. The utilized installation technique in the current work is similar to the installation procedure of thin-foil strain gauges. The geometry of the sensor carrier was utilized to reduce the signal loss. In addition, it improved the ratio between the longitudinal sensitivity and cross-sensitivity. Moreover, the effect of the material properties of the bonding adhesive was evaluated through FEA, which can guide the selection process of the installation adhesive. The piezoresistive behavior of the sensing elements followed very much a linear dependence on strain. It was noticed that at low modulus of elasticity, the effect of layer thickness is minor. However, as the modulus of elasticity increases, *i.e*., the adhesive becomes stiffer; the adhesive layer thickness has major influence. Finally, using FEA it was found that the sensor has low sensitivity to alignment error, e.g., 4.5° rotational error introduced approximately 2% error in the sensor output signal.

## Figures and Tables

**Figure 20.**
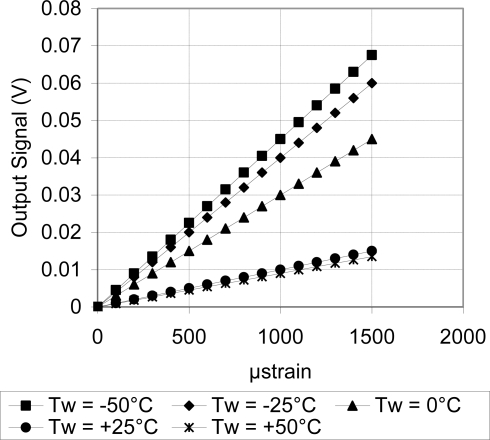
Sensor calibration curves at different operating temperatures for doping concentration of 5 × 10^18^ atoms/cm^3^, V_i_ = 5 V.

**Figure 21.**
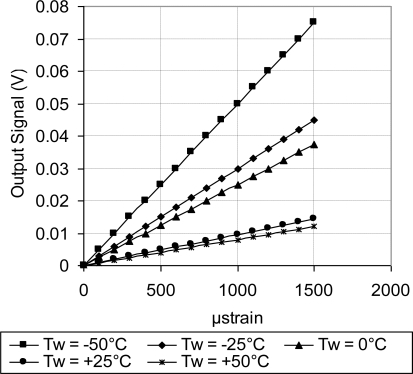
Sensor calibration curves at different operating temperatures for doping concentration of 1 × 10^19^ atoms/cm^3^, V_i_ = 5 V.

**Figure 23.**
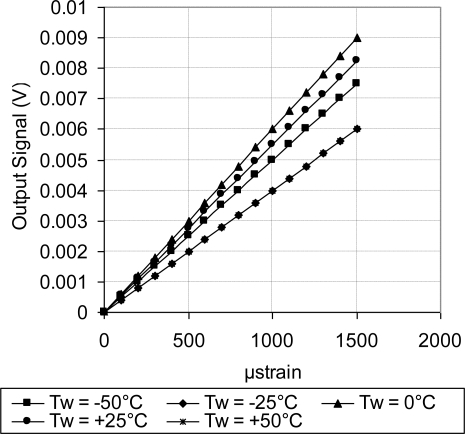
Sensor calibration curves at different operating temperatures for doping concentration of 1 × 10^20^ atoms/cm^3^, V_i_ = 5 V.

**Table 1.** Details of the FEA model.

**Structural Layer**	**Modeling Element**	**Model Input Properties [28]**
Strained surface	*Isotropic*	E = 200 GPa
	20-node tetrahedral elements	ν = 0.33
Bonding layer	*Isotropic*	E = 1 Gpa
	20-node tetrahedral elements	ν = 0.35
Silicon carrier	*Anisotropic*	Elastic constants
	20-node tetrahedral elements	C_11_= 165.7 Gpa
		C_12_= 63.9 Gpa
		C_44_= 79.6 Gpa
Piezoresistors	*Anisotropic*	Elastic constants
	20-node tetrahedral elements with piezoresistive behavior	C_11_= 165.7 Gpa
		C_12_= 63.9 Gpa
		C_44_= 79.6 Gpa
		Piezoresistive Coefficients
		π_11_= 66 Tpa^−1^
		π_12_= −11 TPa^−1^
		π_44_= 1381 TPa^−1^

**Table 2.** TCR values from the published literature and criterion shown on Figure 18.

**Reference**	**Doping Level (atoms/cm^3^)**	**TCR (ppm/°C)**
Figure 1 in Reference [20]	1 × 10^18^	1,900
Figure 1 in Reference [20]	2 × 10^18^	1,000
Figure 1 in Reference [20]	5 × 10^18^	250
Figure 22 in Reference [22]	2 × 10^18^	940
Figure 9 in Reference [30]	1 × 10^16^	2,689
Figure 9 in Reference [30]	3 × 10^18^	2,300
Figure 9 in Reference [30]	9 × 10^18^	889
Figure 9 in Reference [30]	5 × 10^19^	1,187
Figure 9 in Reference [30]	2.7 × 10^20^	1,474
Figure 9 in Reference [30]	2 × 10^21^	1,667
Figure 5 in Reference [37]	1.5 × 10^18^	1,802
Table 3 in Reference [42]	1.1 × 10^18^	2,699
Table 3 in Reference [42]	1.25 × 10^19^	561
Figure 6 in Reference [43]	2 × 10^19^	1,600

**Table 3.** Standard deviation of the calculated sensor sensitivity at different operating temperatures depicted from Figure 24.

**Doping Level (atoms/cm^3^)**	**Standard Deviation in Sensor Sensitivity (mV/με)**				
	**−50 °C**	**−25 °C**	**0 °C**	**25 °C**	**50 °C**

**1 × 10^18^**	1.51 × 10^−03^	3.17 × 10^−06^	4.40 × 10^−06^	1.48 × 10^−03^	1.41 × 10^−03^
**5 × 10^18^**	1.49 × 10^−03^	2.16 × 10^−06^	1.46 × 10^−06^	1.48 × 10^−03^	1.42 × 10^−03^
**1 × 10^19^**	1.44 × 10^−03^	5.99 × 10^−06^	4.04 × 10^−06^	6.08 × 10^−04^	5.93 × 10^−04^
**5 × 10^19^**	2.81 × 10^−04^	7.85 × 10^−07^	1.89 × 10^−06^	2.90 × 10^−04^	2.93 × 10^−04^
**1 × 10^20^**	3.08 × 10^−04^	4.20 × 10^−06^	6.93 × 10^−07^	3.09 × 10^−04^	3.01 × 10^−04^
